# Targeted activation of Nrf2/HO‐1 pathway by Corynoline alleviates osteoporosis development

**DOI:** 10.1002/fsn3.3239

**Published:** 2023-01-24

**Authors:** Tian‐hao Xu, Bing‐hao Lin, Cheng‐bin Huang, Jing‐yu Sun, Kai Tan, Run‐xun Ma, Yi‐xun Huang, She‐ji Weng, Wen‐lai Fang, Wei‐kai Chen, Bing‐li Bai

**Affiliations:** ^1^ Department of Orthopaedic The Second Affiliated Hospital and Yuying Children's Hospital of Wenzhou Medical University Wenzhou China; ^2^ Key Laboratory of Orthopaedics of Zhejiang Province Wenzhou China; ^3^ School of Medicine Shanghai University Shanghai China

**Keywords:** Corynoline, reactive oxygen species (ROS), osteoblast, Nrf2/HO‐1 pathway

## Abstract

Oxidative stress is preferentially treated as a risk factor for the development and progression of osteoporosis. Corynoline as a component of Corydalis bungeana Turcz presents antioxidative and anti‐inflammatory properties. In the present study, the effects of Corynoline on osteoblasts following hydrogen peroxide (H_2_O_2_)‐induced injury were evaluated accompanied by the investigation of the molecular mechanisms involved. It was found that Corynoline downregulated the intracellular reactive oxygen species (ROS) generation and restored the osteogenic potential of the disrupted osteoblasts by H_2_O_2_ exposure. Furthermore, Corynoline was revealed to activate the Nrf2/HO‐1 signaling pathway, while ML385 (an Nrf2 inhibitor) would prevent the Corynoline‐mediated positive effects on the disrupted osteoblasts. In terms of the animal experiments, Corynoline treatment contributed to a significantly alleviated bone loss. These findings indicate that Corynoline may significantly attenuate the H_2_O_2_‐induced oxidative damage of osteoblasts via the Nrf2/HO‐1 signaling pathway, providing novel insights to the development of treatments for osteoporosis induced by oxidative injury.

## Graphical abstract

Corynoline could upregulate the antioxidative enzyme expression in H2O2‐treated osteoblasts by activating the Nrf2 pathway, reducing the negative effect of oxidative stress, and strengthening the function of osteoblasts. The in vitro experiment results also showed that Corynoline delayed osteoporosis progression in the rat model and restored Nrf2 in situ expression. Overall, Corynoline could activate the Nrf2 pathway and mitigate or suppress osteoporosis‐related bone loss through the Nrf2 pathway, showing promise as a potential treatment for osteoporosis.

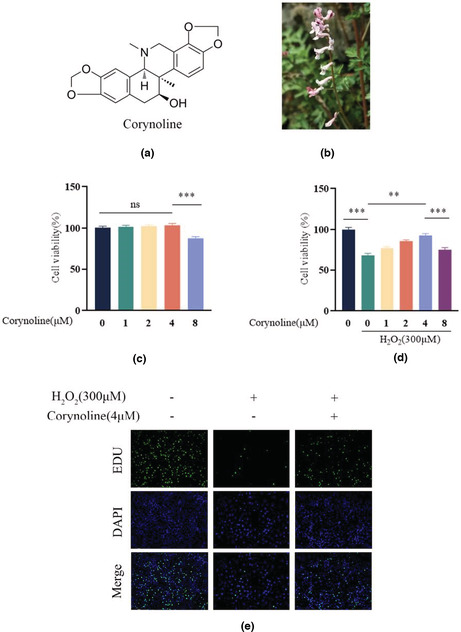

## 1 INTRODUCTION

Osteoporosis will lead to an increased risk of fractures (Kuek et al., 2016). With the aging populations, osteoporotic fractures will aggravate the burden on society in the coming years (Sahni et al., 2009), which has become a major public health issue. Among the numerous factors, the increased level of ROS lies one of the essential causes of osteoporosis, which could alleviate the osteoblastic differentiation of osteoblasts (Lee et al., 2021; Liu, Mao, et al., 2017). One study has reported that the significantly upregulated levels of oxidative stress‐related biomarkers, while the decreased antioxidant levels in the serum of postmenopausal women with osteoporosis (Xiao et al., 2020). Hence, modulating ROS production can be a potential strategy for osteoporosis therapies.

Nuclear factor erythroid‐2‐related factor 2 (Nrf2) is considered a primary regulatory factor for antioxidant resistance (Do et al., 2021). Since the accumulated ROS will accelerate the development of osteoporosis while the activated Nrf2 can effectively reduce oxidative stress, it could serve as a promising therapeutic target for osteoporosis. Previous studies demonstrated the significantly downregulated bone mass and bone strength in Nrf2‐deficient mice compared to wild‐type mice (Park et al., 2020). Furthermore, heme oxygenase‐1 (HO‐1), the downstream protein of Nrf2, exhibits the potential to clear ROS and the antioxidative stress (Su et al., 2020). Overall, the Nrf2/HO‐1 signaling pathway is considered the efficient cytoprotective defense mechanism against ROS‐induced cell damage.

Plants can be adopted as potential sources of natural antioxidants (Sunthonkun et al., 2019). For example, Corynoline, derived from Corydalis bungeana Turcz, has been reported to exhibit multiple pharmacologic properties. Li et al. (2022) demonstrate this component to alleviate osteoarthritis via the Nrf2/NF‐kB pathway. In addition, Wu et al. reported that it could protect lipopolysaccharide‐induced mastitis by manipulating the Nrf2 signaling pathway (Wu et al., 2021). However, the specific mechanism that Corynoline ameliorates oxidative stress‐induced damage remains unclear. In the present study, H_2_O_2_ was used to induce oxidative stress in osteoblasts, as previously reported. We demonstrated for the first time that Corynoline could suppress H_2_O_2_‐induced osteoblast damage by activating the Nrf2/HO‐1 pathway, proving Corynoline as a suitable therapeutic option for osteoporosis.

## 2 MATERIALS AND METHODS

### 2.1 Antibodies, reagents, and media

Corynoline (≥96%) and H_2_O_2_ were provided by Sigma‐Aldrich, and the RUNX2, COL1A1, and BMP2 primary antibodies were provided by Abcam, while the HO‐1, Nrf2, and GAPDH from Cell Signaling Technology. The penicillin/streptomycin, Dulbecco's Modified Eagle Medium (DMEM), and fetal bovine serum (FBS) were purchased from Gibco BRL provided.

### 2.2 Isolation of primary osteoblasts

The calvarial bone obtained from the 1‐day‐old SD rats was digested by trypsin‐collagenase to primary osteoblasts, which were then cultured in DMEM enriched with 10% (v/v) FBS and 1% (v/v) penicillin–streptomycin at 37°C under 5% CO_2_. No‐adherent osteoblasts after 12 h of incubation were removed, whereas the adherent cells were cultured in fresh media for follow‐up experiments, which were changed every two to three days.

### 2.3 Cell culture

When the cell confluence reached about 80%–90%, the medium was freshened every two days, with the cells passaged. Afterwards, the cells were divided into three groups for different treatments: (Kuek et al., 2016) The control group referred to the untreated osteoblasts cultured for a similar period as the other groups; (Sahni et al., 2009) the H_2_O_2_ group referred to cells treated with 300‐μM H_2_O_2_ for one day before cultured for two full days in a complete DMEM medium; (Lee et al., 2021) the H_2_O_2_ + Corynoline group referred to cells treated with 300‐μM H_2_O_2_ for one day before cultured for two full days in 4‐μM Corynoline.

### 2.4 Cell counting kit‐8 (CCK‐8) proliferation assay and 5‐ethynyl‐20‐deoxyuridine (EdU) incorporation assay

The CCK‐8 assay (Beyotime) was performed to determine the Corynoline and H_2_O_2_ effect on osteoblast vitality. The cells were seeded at 5 × 10^3^ cells/well in a 96‐well plate for two full days with different Corynoline concentrations (1, 2, 4, and 8 μM) in the absence or presence of H_2_O_2_ pretreatment, then incubated for another 60 min after 10 μl of CCK‐8 reagent added to each well. A Multiskan GO microdisk spectrophotometer (ThermoFisher Science) was utilized to measure the optical density (OD) or absorbance at 450 nm.

The cells were proliferated using the EdU Cell Proliferation Kit (Beyotime) (Ni et al., 2022). Briefly, osteoblasts were incubated with EdU for 4 h and fixed with 4% polyformaldehyde for 30 min. After washing with PBS, the Hochest was taken for nuclear staining. Finally, the stained cells were observed and photographed with a microscope.

### 2.5 Osteogenic differentiation and mineralization

The cells were seeded at 5 × 10^4^ cells/well in a 24‐well plate. After being treated with H_2_O_2_ and Corynoline, the cells were cultured in DMEM with 10 mm of β‐glycerol phosphate and 20‐μM ascorbic acid. The media were freshened every other day. The ALP activity was detected using alkaline phosphatase (ALP) staining kit (Beyotime) seven days after differentiation. The cells were then cultured for three weeks under osteogenic conditions, fixed, and stained with alizarin red (ARS) (Beyotime).

### 2.6 Western blotting

The cells were lysed in RIPA lysis buffer (Beyotime). In addition, the nuclear and cytoplasmic protein components were isolated using the commercial kit following the manufacturer's instructions (Chen et al., 2020). After cell lysate centrifugation, the supernatant protein concentration was determined according to BCA protein analysis (Beyotime). The same amounts of protein were extracted by 10% SDS‐PAGE and transferred onto a polyvinylidene difluoride (PVDF) membrane (Schwalbach). The blocked protein was incubated with 5% skim milk powder and primary antibodies at 4°C for 12 h. Subsequently, the membrane was washed with TBST for three times and incubated at room temperature with the corresponding HRP binding secondary antibody for 4 h. The positive bands were visualized on ChemiDoc XRS + imager (bio RAD) by employing the enhanced chemiluminescence, followed by the quantification of positive bands through a densitometry analysis on the image lab v3.0 software (bio RAD). The protocol was repeated for three times.

### 2.7 Reactive oxygen species assay

The dihydroethidium (DHE) test (Yeasen Biotech) based on a fluorescent probe were performed to quantify the intracellular ROS levels. The treated osteoblasts were washed with PBS for three times then incubated with DHE at 37°C for 30 min in darkness. The cells were immediately turned to the observation under a microscope (Olympus Life Science). Photos were randomly captured for three samples for analysis using the Image‐Proplus software version 6.0.

### 2.8 Mitochondrial function assays

The JC‐1 probes (Yeasen Biotech) and MitoSox (ThermoFisher Science) were employed to determine the osteoblast MMP (mitochondrial membrane potential) and the levels of superoxide ions following the manufacturer's guidelines (Chen et al., 2020). The stained cells were observed under a fluorescence microscope (Olympus Life Science).

### 2.9 Quantification of the activities of GPX and CAT

The cells were washed with PBS twice were lysed for 30 min on ice. Commercial assay kits (Jiancheng Biotechnology) were employed to detect the GPX and CAT activities in the lysates, with the manufacturer's instructions followed (Chen et al., 2020).

### 2.10 Molecular docking

Corynoline and Nrf2 were chosen for molecular docking research. The compound, molecular weight, and 2D structure of the Cor were determined from the PubChem database. The crystal structure of Nrf2 (PDB ID: 2flu) was obtained from the PDB database (https://www.rcsb.org/). Before docking, AutoDock software (http://vina.scripps.edu/) was used to preprocess the ligand and protein structures. Briefly, the structures were mainly subjected to removal of water molecules, hydrogenation, modification of amino acids, optimization of energy and adjustment of force field parameters, and then molecular docking. The Binding Affinity (kcal/mol) value represents the binding ability of the ligand and protein. The lower the binding ability, the more stable the binding between the ligand and protein. Finally, the 3D and 2D docking images were presented by UCSF PyMoL and ligplus softwares, respectively.

### 2.11 Immunofluorescence (IF).

After treated, the osteoblasts were fixed with 4% paraformaldehyde (PFA) for 15 min at room temperature and immersed in PBS for 20 min with 0.5% (V/V) Triton X‐100. Subsequently, the cells were incubated with 1% goat serum for 1 h at room temperature to block non‐specific antibody binding sites, then incubated at 4°C overnight with primary antibodies, rinsed with PBS for three times, and further incubated with fluorescence‐conjugated secondary antibodies at room temperature for 1 h in the dark. After the cell nuclei stained with DAPI for 5 min at room temperature, the cells were visualized under a fluorescent microscope (Olympus Life Science). Finally, the quantitative analysis of IF results was performed on Image‐Pro Plus 2D software (Rockville).

### 2.12 Animal model and treatment

All the animal experiments were approved by the Wenzhou Medical University Second Affiliated Hospital and Yuying Children's Hospital's Ethics Committee (Approved number: XMSQ2022‐0524) and were also carried out in accordance to the guideline standards for the experimental animals' care and use (National Research Council Committee for the Update of the Guide for the C, Use of Laboratory A, 2011). Forty‐five 2‐month‐old female SD rats provide by the Shanghai Experimental Animal Center provided were raised at 22°C–25°C and 12‐hour light/dark cycles under specific pathogen‐free conditions with tap water and food ad libitum. The standard diet provided 70%–80% carbohydrates, 5% fat, 1% calcium, 2.5% casein, and 0.8% phosphorus. One week after domestication, the rats were randomly assigned to sham, Ovariectomized (OVX), and OVX + Corynoline groups (*n* = 15 in each group). Bilateral OVX was utilized in thirty rats using the double dorso‐lateral method described by Chen et al, which were randomly assigned to the OVX and OVX + Corynoline groups (*n* = 15 in each group) after 8‐week intervention. The OVX + Corynoline group was administrated with 10 mg/kg of Corynoline diluted with saline solution for 8 weeks. Finally, they were sacrificed for the extraction of their bilateral femurs that were treated with 4% paraformaldehyde. All the procedures were strictly met with the Wenzhou Medical University's animal care and use committee requirements.

### 2.13 Micro‐CT analysis

The microstructure of distal femur was analyzed on a cabinet cone‐beam micro‐CT system and software (μCT 50) at 70 kV and 200 kVμA. Images were collected at 14.8‐mm Omni‐directional spatial resolution to generate the 3D reconstructions. The volume of interest (VOI) referred to the trabecular chamber 2 mm beneath the growth plate's highest point and contained 100 CT slices at the distal end. The assessed quantitative bone parameters in VOI involved bone volume‐to‐tissue volume (BV/TV) percentage, mean trabecular thickness (TB. Th, mm), mean trabecular number (TB. N, 1/mm), and mean trabecular separation (TB. SP, mm).

### 2.14 Continuous fluorescent labeling

Continuous fluorescent labeling was performed on rat femurs. In four and six weeks after receiving Corynoline, respectively, the three groups of rats were administrated with calcein by injected intraperitoneally. The epiphysis of femoral shaft tissue samples was harvested for hard‐tissue slicing and imaged under a fluorescence microscope.

### 2.15 Immunohistochemistry (IHC)

Each femur was fixed by calcified in 10% EDTA for one month, dehydrated with ethanol gradient (70%–100%), removed by xylene, and embedded in paraffin. According to the manufacturer's instructions, hematoxylin‐eosin (HE) and Masson's trichrome staining (Solarbio Science & Technology) were performed in a longitudinal direction on 4‐μm‐thick serial sections. Afterwards, the slices were treated with primary antibodies against Nrf2 and COL1A1, rinsed with PBS for three times, and incubated with horseradish peroxidase‐conjugated secondary antibodies. The expression levels of Nrf2 and COL1A1 were analyzed on the Image‐Pro Plus software.

### 2.16 Statistical analysis

GraphPad Prism software was employed in all statistical analyses. The experiment results repeated at least three times were expressed as the mean ± mean standard error (SEM). The two groups' mean values were compared by performing the two‐tailed Student's *t*‐test, and multiple comparisons by one‐way ANOVA and Bonferroni or Dunnett's correction. *p* < .05 was considered statistically significant.

## 3 RESULTS

### 3.1 Effects of Corynoline on cell viability and proliferation

**FIGURE 1:**
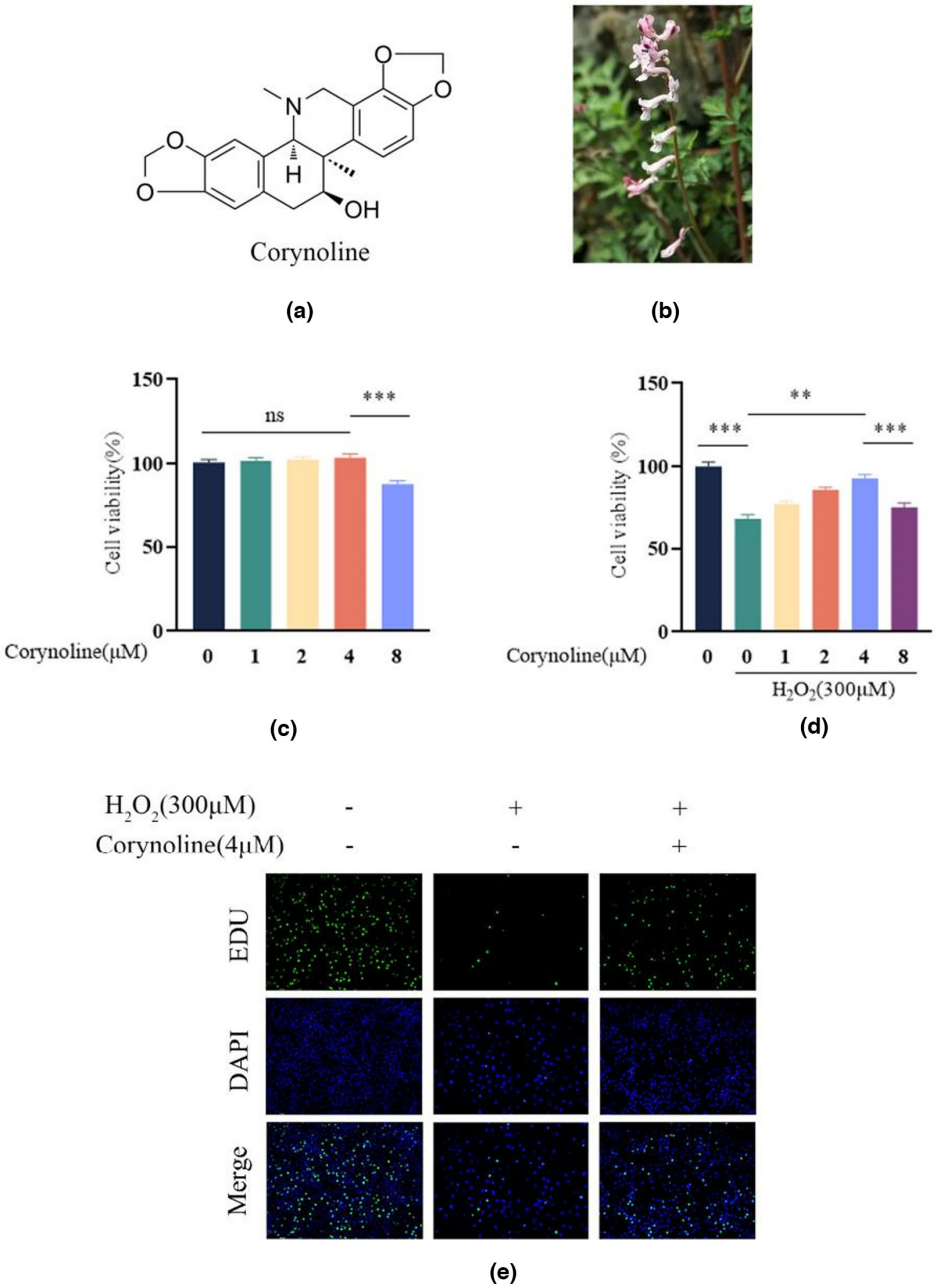
Effects of Corynoline on osteoblast viability. (a) The chemical structure of Corynoline. (b) The field photograph of Corynoline. (c) Percentage of viable cells after being treated with different Corynoline doses for 48 h. (d) Percentage of viable cells pretreated with H_2_O_2_ with/without different concentrations of Corynoline. (e) Representative fluorescence images were displayed the reduced effect of H_2_O_2_ by Corynoline culture on osteoblast proliferation. The data in the figures represent the averages ± SEM of three times in duplicates. **p < .01, ***p < .001 versus the relative groups

Chemical structure of Corynoline (Figure 1a). Photograph of representative plants (Figure 1b). According to the previous reports, 300‐μM H_2_O_2_ was employed in osteoblasts but not in the control cells (Squillaro et al., 2019). As the CCK‐8 assay revealed, the low levels of Corynoline exerted a promising role in cell growth, whereas high levels exerted the opposite (Figure 1c). 4‐μM Corynoline was utilized for subsequent experiments based on CCK‐8 outcomes. The decreased osteoblast counts resulting from H_2_O_2_ treatment could be reversed by Corynoline (Figure 1d). Corresponding to these findings, EdU staining revealed the decreased osteoblast proliferation by H_2_O_2_ could be attenuated by employing Corynoline (Figure 1e).

### 3.2 Corynoline mitigated H_2_O_2_ ‐induced oxidative stress in osteoblasts

**FIGURE 2:**
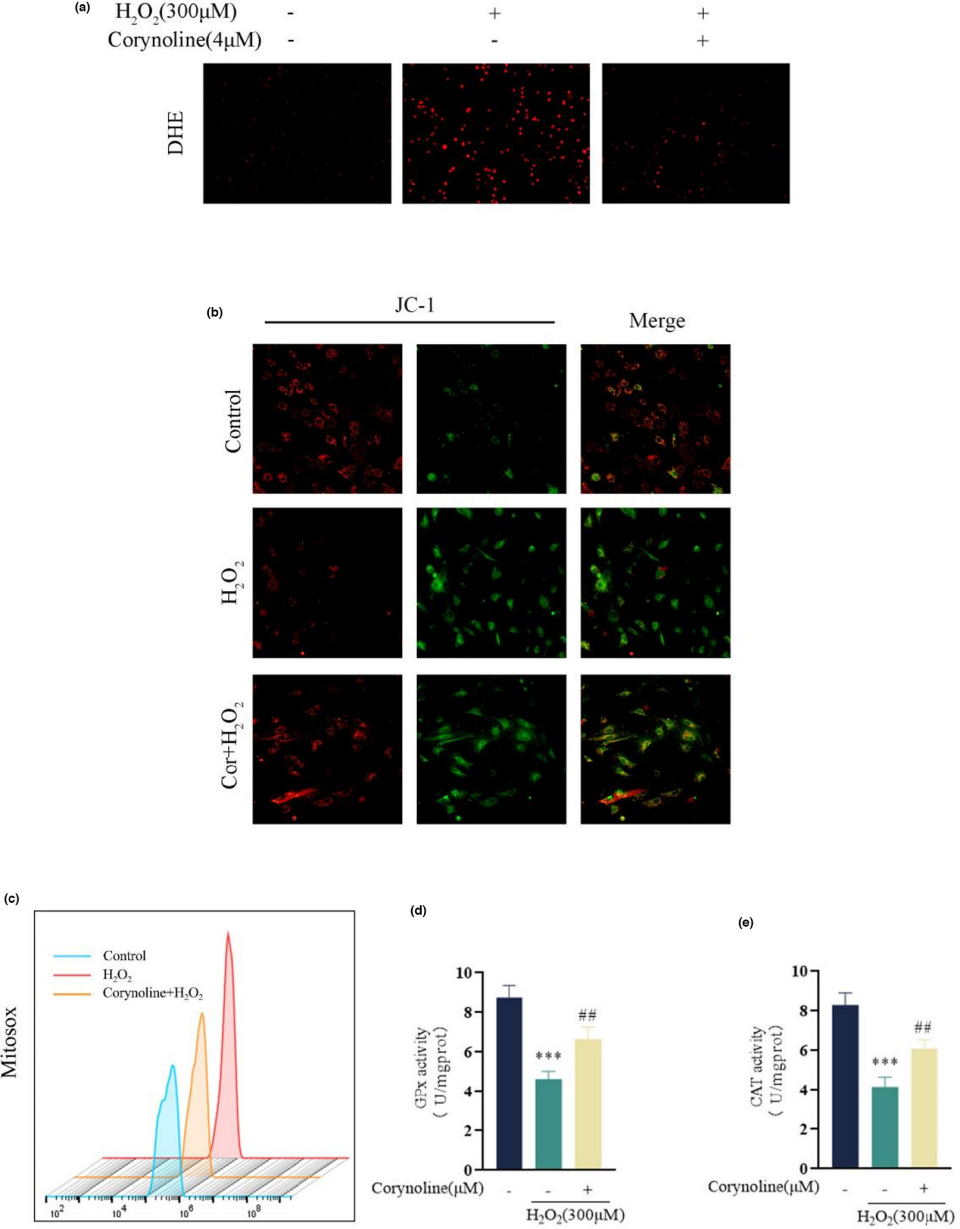
Corynoline neutralized H_2_O_2_‐induced oxidative stress and mitochondrial dysfunction in osteoblasts. (a) Representative images for the DHE level in the differentially treated osteoblasts. (b) Representative images for JC‐1 intensities in the differentially treated osteoblasts. (c) The relative mean fluorescence intensity of MitoSox was measured by flow cytometry. (d‐e) The activities of GPx and CAT in different groups. The data in the figures represent the averages ± SEM of three times in duplicates. ***p < .01 versus the untreated group, ^##^ *p* < .01 versus the H_2_O_2_ treated group

One of the etiologic factors for osteoporosis is the damaged osteogenic capability due to the high oxidative stress levels. As a result, the impact of Corynoline on intracellular ROS production was assessed here, accompanied by the levels of mitochondrial ROS, and the mitochondrial function in H_2_O_2_‐treated osteoblasts. The treatment with H_2_O_2_ markedly increased the DHE in comparison to the control group, which was reversed by Corynoline, and returned close to the normal level (Figure 2a). MitoSox, a specific dye for mitochondrial ROS, was adopted to detect mitochondrial ROS, which showed that Corynoline treatment reduced MitoSox intensity compared to the H_2_O_2_ group according to the flow cytometric analysis of it (Figure 2c). JC‐1 probes were employed to detect MMP. H_2_O_2_‐treated cells exhibited significantly decreased MMP compared to the control group, but returned to near‐physiological levels after being treated by Corynoline (Figure 2b). Additionally, the GPX and CAT activities were restored in osteoblasts co‐cultured with H_2_O_2_ and Corynoline (Figure 2d,e).

### 3.3 Corynoline reduced the H_2_O_2_ inhibitory effect on osteoblast differentiation and mineralization

**FIGURE 3:**
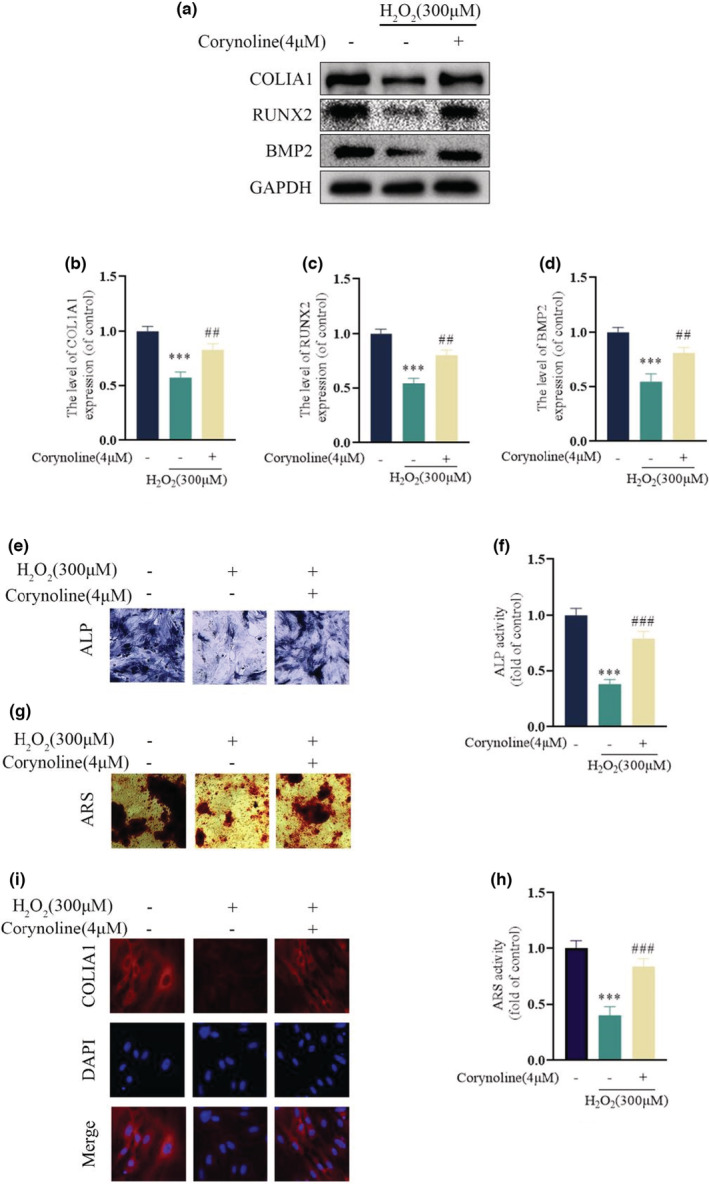
Corynoline restored differentiation and mineralization of H_2_O_2_‐treated osteoblast. (a–d) Expression levels of COL1A1, RUNX2, and BMP2 in the differentially treated osteoblasts. (e, f) Representative images for ALP staining on the 7th day and (g, h) ARS staining on the 21st day of osteogenic induction in differentially treated osteoblasts. (i) Representative images for fluorescence intensity of COL1A1 in the differentially treated cells. Data are described as the average ± SEM of three independent experiments. ***p < .001 versus the untreated group. ^##^ *p* < .01, ^###^ *p* < .001 versus the H_2_O_2_ treated group

Oxidative stress inhibition exerted a vital influence on the osteoblast osteogenic function. Herein, we investigated the impact of Corynoline on the early differentiation and mineralization of H_2_O_2_‐treated cells. Determined by ALP activity after seven days of culturing, H_2_O_2_ significantly reduced the osteogenic differentiation and the calcium nodules formation on the 21st day (Figure 3e‐h). However, the group treated with Corynoline restored their ALP activity and mineralization, which was consistent with the up‐regulated osteogenic transcription factors COL1A1, Runx2, and BMP2 after seven days of osteogenesis induction (Figure 3a–d). In addition, fluorescence images revealed the reduced COL1A1 expression in osteoblasts by H_2_O_2_, which could be neutralized by Corynoline (Figure 3i). In conclusion, Corynoline could prevent the negative impacts of H_2_O_2_ on osteoblasts' osteogenic differentiation.

### 3.4 Corynoline exerted its protective effect against H_2_O_2_ via the Nrf2/HO‐1 signaling pathway

**FIGURE 4:**
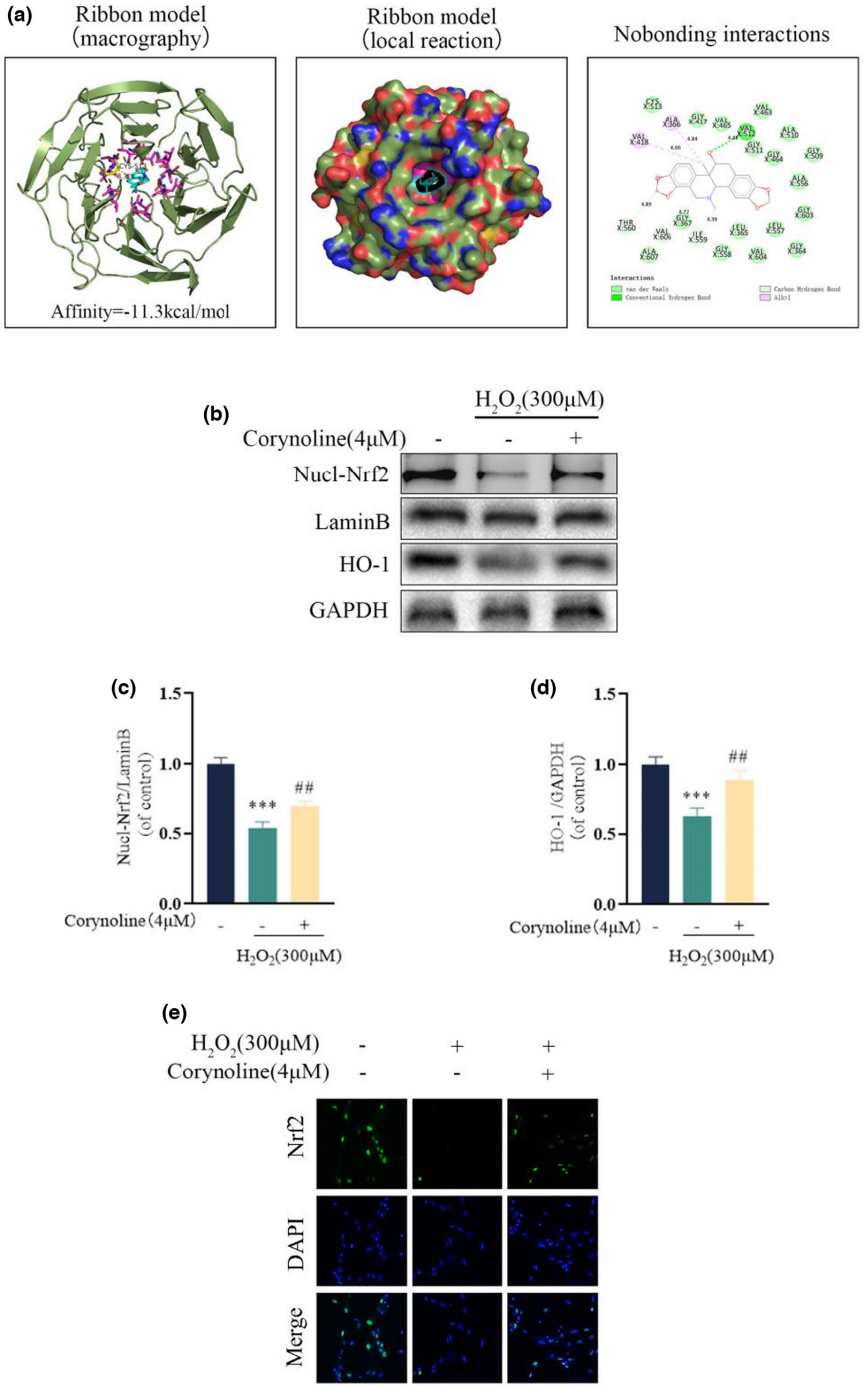
Corynoline activated the Nrf2/HO‐1 pathway in osteoblasts. (a) A ribbon model is applied to represent protein residues and illustrates a 3D binding model. Binding site affinity = −11.2 kcal/mol exists in Corynoline docking with Nrf2. (b–d) Expression levels of Nucl‐Nrf2 and HO‐1 in the differentially treated osteoblasts. (e) Representative fluorescence images for Nrf2 localization in the differentially treated cells. The data in the figures represent the averages ± SEM of three times in duplicates. ***p < .001 versus the untreated group, ^##^ *p* < .01 versus the H_2_O_2_ treated group

**FIGURE 5:**
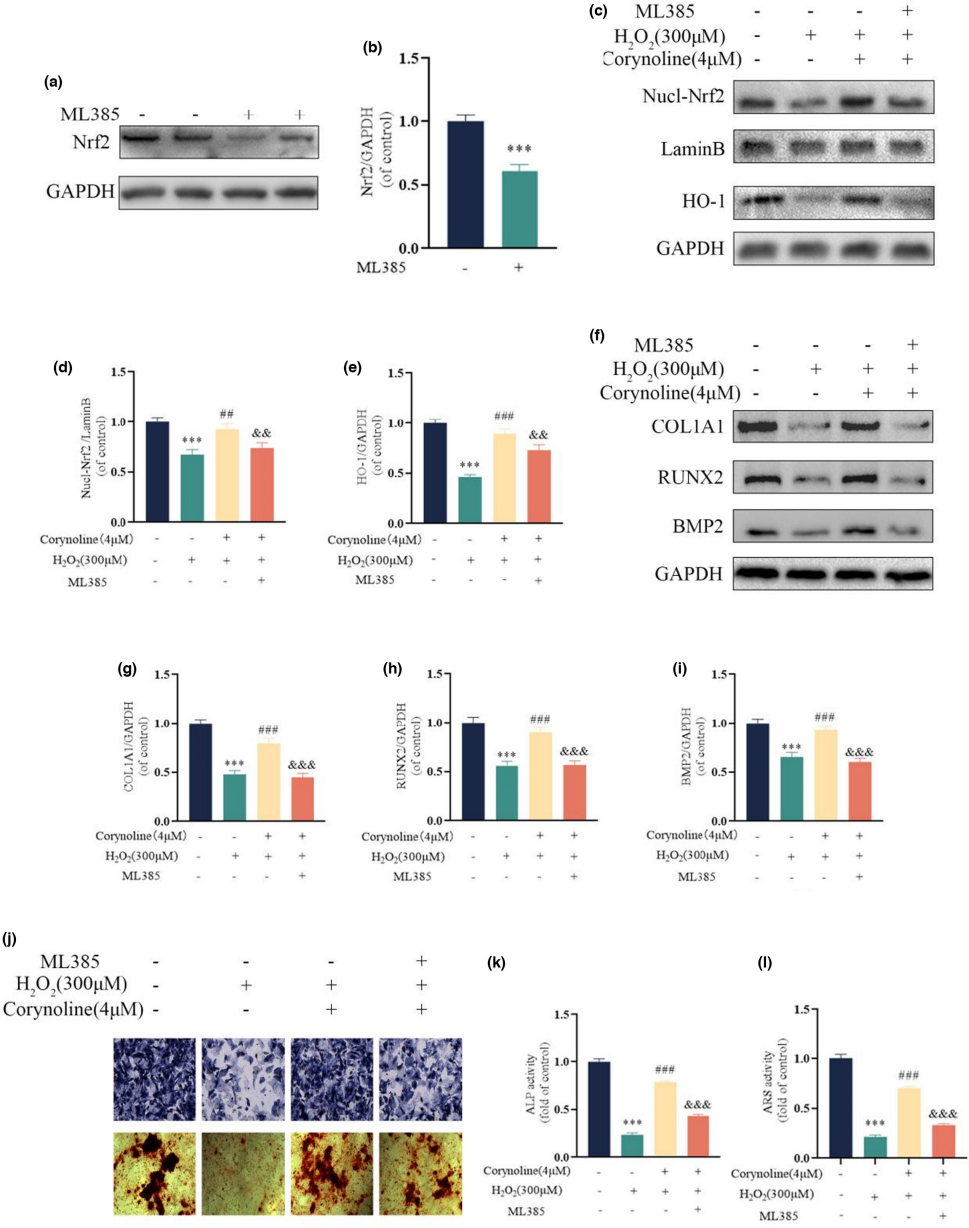
The Nrf2 pathway mediated the osteoprotective effects of Corynoline. (a, b) Western blotting for assessing the expression of Nrf2 after ML385 treatment. (c–e) Immunoblot of Nucl‐Nrf2 and HO‐1 in the differentially treated osteoblasts. (f–i) Immunoblot of COL1A1, RUNX2, and BMP2 in the differentially treated osteoblasts. (j–l) Representative images of ALP staining on the 7th day and ARS staining on the 21st day of osteogenic induction in differentially treated BMSCs. The data in figures represent the averages ± SEM of three times in duplicates. ***p < .001 versus the untreated group; ^##^ *p* < .01, ^###^ *p* < .001 versus the H_2_O_2_ treated group; ^&&^ *p* < .01, ^&&&^ *p* < .001 versus the Corynoline group

To examine the affinity of Corynoline and Nrf2, the molecular docking was simulated on an artificial intelligence software, with the Corynoline chemical structure employed for further analysis. Upon all the established possible models, it was found that Corynoline was stabilized in the binding channel primarily by hydrophobic interaction and hydrogen bond, which formed a hydrogen bond with VAL512 accompanied by a hydrophobic interaction with ALA366 and VAL418. The average binding energy between the Corynoline and Nrf2 was all measured at ‐11.3 kcal/mol (Figure 4a). Western blotting revealed the downregulated nuclear‐Nrf2 and HO‐1 in H_2_O_2_‐treated cells, which could nearly return to normal levels after treated with Corynoline. Corynoline reversed the inhibitory effect of H_2_O_2_ on HO‐1 expression and Nrf2 nuclear translocation (Figure 4b–d). In addition, fluorescence images showed the reduced nuclear translocation of Nrf2 in osteoblasts by H_2_O_2_, which was neutralized by Corynoline (Figure 4e). For a more in‐depth exploration on the mechanism of Corynoline, we blocked the activation of the pathway by inhibiting the Nrf2 expression using ML385. Notably, the suppression of Nrf2 inhibited the protective capability of Corynoline against H_2_O_2_. The western blotting showed the significantly reduced levels of Nrf2 expression resulting from ML385 (Figure 5a–e). Seven days after osteogenic induction, the expression levels of COL1A1, RUNX2, and BMP2 in osteoblasts treated with Corynoline were partially downregulated by ML385, with beneficial impacts on osteoblasts' mineralization and differentiation from Corynoline reserved (Figure 5f–l). These findings demonstrated the way that Corynoline induced nuclear translocation of Nrf2 and activated its downstream pathways to exert its efficacy.

### 3.5 Corynoline treatment decreased osteoporosis development in vivo

**FIGURE 6:**
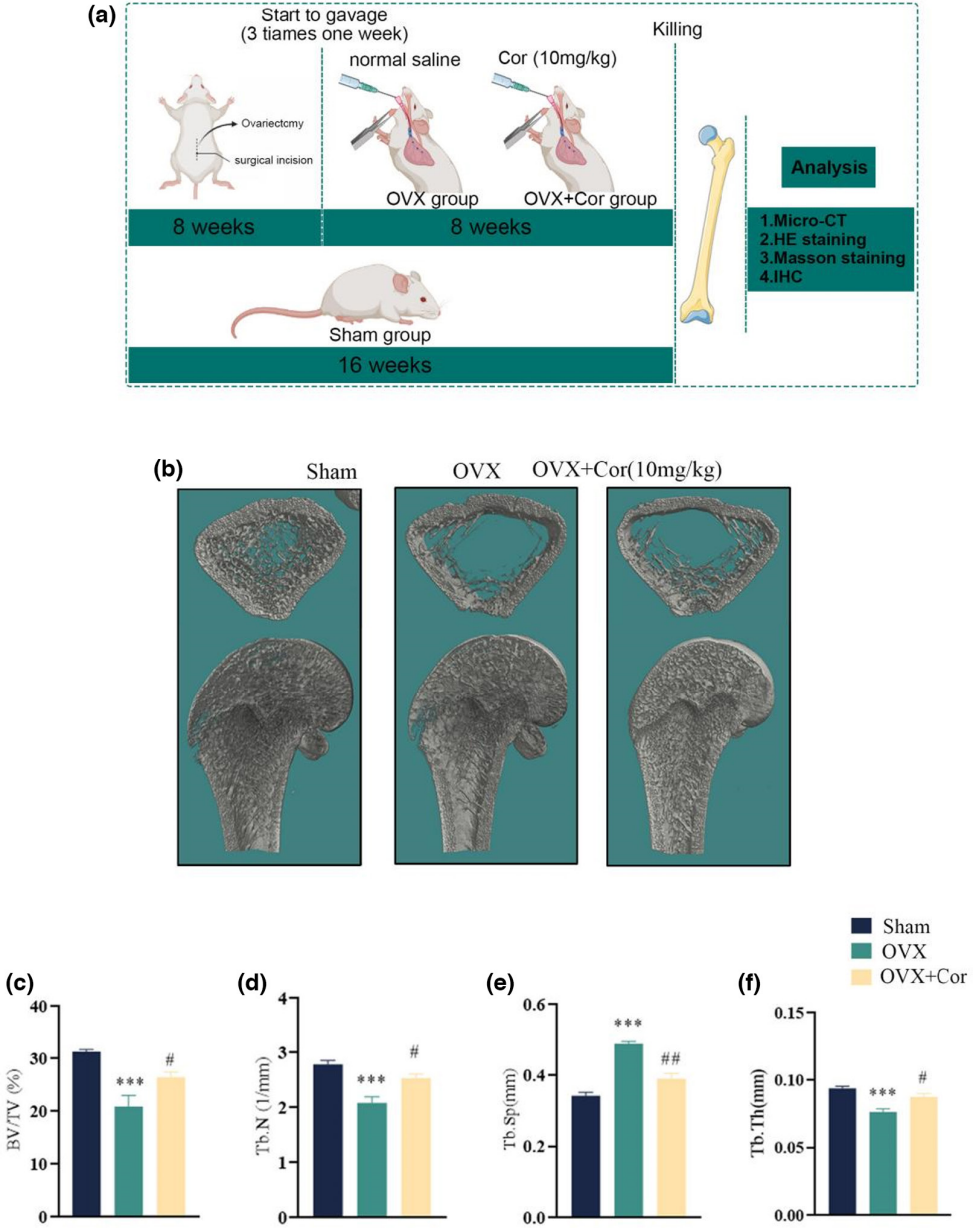
Corynoline treatment alleviated bone microarchitecture and mass. (a) The treatment and administration process of rats. (b) Representative micro‐CT images of the longitudinal and transverse sections of the distal femurs. (c–f) The BV/TV, Tb.Sp, Tb.N, and Tb.Th values in the differentially treated animals. Data are expressed as averages ± SEM of five times in duplicates. ***p < .001 versus the Sham group; ^#^ *p* < .05, ^##^ *p* < .01 versus the OVX group

**FIGURE 7:**
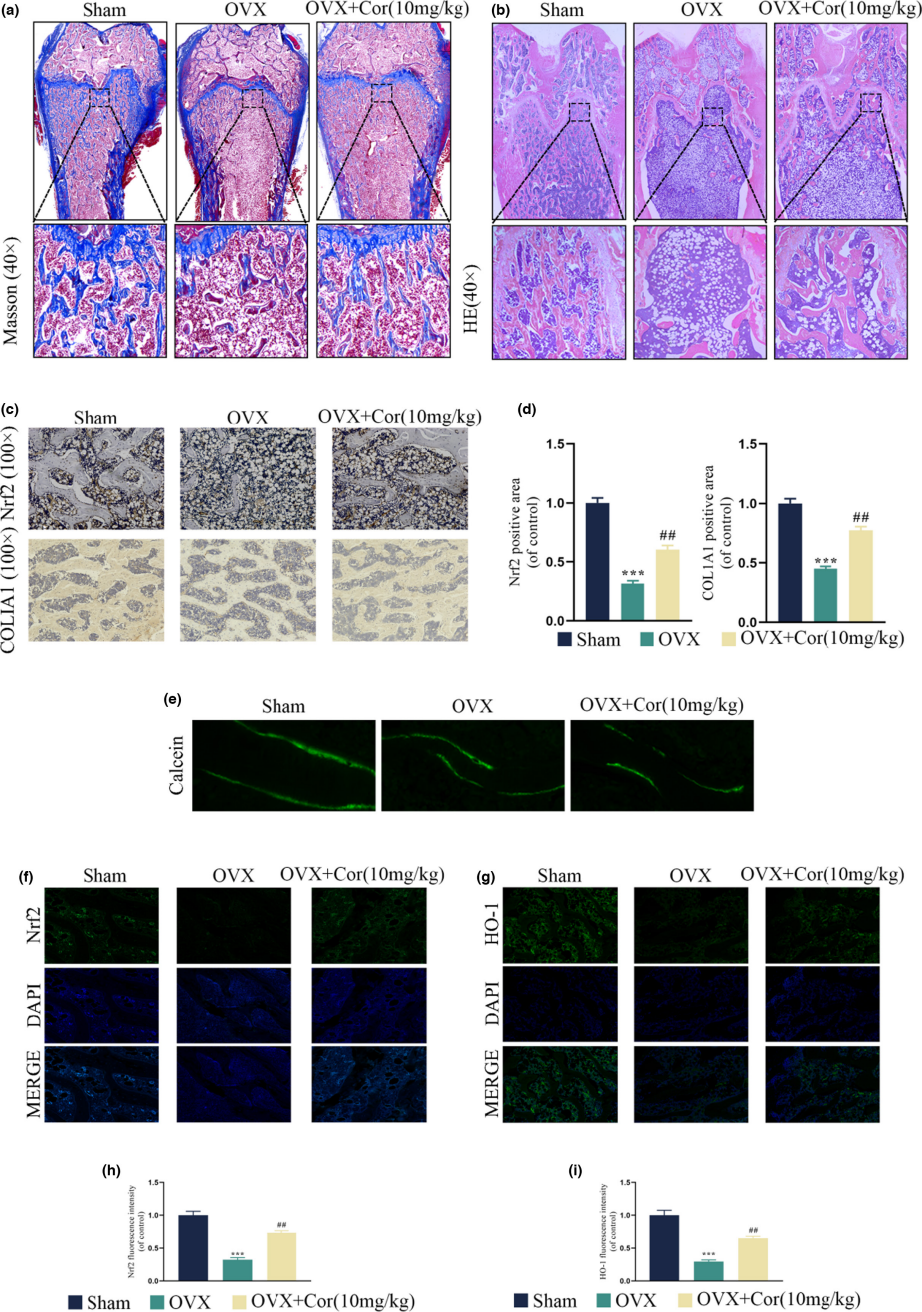
Corynoline treatment revived the microstructure and augmented bone formation of the rats. (a, b) Representative images of H&E staining and Masson's staining in different groups. (c, d) Nrf2, COL1A1 immunohistochemical staining, and quantitative analysis of the metaphyseal tissue of the thigh in different groups. (e) New bone formation was detected according to sequential fluorescent labeling of calcein. (e–i) Nrf2, HO‐1 immunofluorescence staining, and quantitative analysis of the metaphyseal tissue of the thigh in different groups. Data are expressed as averages ± SEM of five times in duplicates.

The postmenopausal osteoporosis rat model was adopted in this research. The workflow of animal experimental is detailed in Figure 6a. To determine the distal femur microstructure, the micro‐CT scanning and other parameters in all groups were calculated, covering BV/TV, Tb.Th, Tb.Sp, and Tb.N. Compared to the untreated osteoporotic rats, the Corynoline treatment for 8 weeks upregulated the BV/TV, Tb.N, Tb.Th and downregulated Tb.Sp. The distal femur histological sections also revealed the increased number of trabeculae in the postmenopausal osteoporosis model treated with Corynoline compared to the untreated postmenopausal osteoporosis model (Figure 6b–f and Figure 7a,b). Correspondingly, the Nrf2 and COL1A1 expression of distal femur was higher in the Corynoline‐treated group in comparison to the untreated osteoporotic rats (Figure 7c). Based on the calcein‐labeled bone mineralization process four and six weeks after Corynoline treatment, the broadness of distance strip in the Corynoline group (marked by green) was elevated compared to the postmenopausal osteoporosis group (Figure 7d). Finally, Nrf2 and HO‐1 levels were significantly elevated in Cor‐treated distal femurs compared to OVX‐treated distal femurs (Figure 7e,f). In conclusion, Corynoline still exerted a potential therapeutic effect on postmenopausal osteoporosis.

## 4 DISCUSSION

Osteoporosis emerges as a bone metabolic disease with progressive development that affects a prominent group of people worldwide, especially for postmenopausal women characterized by bone mass reduction and deterioration of osseous microarchitecture, which elicit the degenderized bone strength and elevated risk of fragility fractures, bringing the high morbidity, mortality, and medical costs (Khan & Khan, 2008; Yeh et al., 2020). Postmenopausal osteoporosis will typically occur about 5–10 years after menopause due to the decreased estrogen levels (Yeh et al., 2020). However, a long‐term preventive and curative medical care still remains lacking currently. Corynoline is a component of corydalis bungeana Turcz and brings lighter side effects in comparison to the currently available osteoporotic treatments, which is expected to promise as a superior treatment modality for this disease (Li et al., 2022). The present study demonstrated the significantly decreased osteoblast viability and ROS production induced by the administration of H_2_O_2_, while a pretreatment with Corynoline could enhance the cell viability by conferring resistance to oxidative stress‐induced apoptosis. In addition, Corynoline promoted the osteoblast survival and osteogenesis by preventing ROS production induced by H_2_O_2_, upregulating the expression of osteogenic markers, and activating Nrf2/HO‐1 signaling pathways. Furthermore, the increase of Nrf2 expression and reduction of bone loss in the distal femur by Corynoline treatment were confirmed in a postmenopausal osteoporosis rat model.

Corynoline as a natural product is a unique isoquinoline alkaloid extracted from traditional Chinese medicine Corydalis bungeana Turcz. It exerts the protective effects against inflammation‐related diseases, such as upper respiratory tract infections, acute lung injury, and inflammation of the cardiovascular system, of which the antioxidative properties have not been investigated (Liu, Song, et al., 2017; Wei et al., 2021). As a result, we speculated a vital role of Corynoline in the high‐ROS microenvironment in osteoblasts.

Oxidative stress is essential in the pathogenesis of osteoporosis that refers to an imbalance between oxidation mechanisms for ROS generation and the antioxidant mechanisms for ROS scavenging (Xiao et al., 2020). Hence, antioxidant therapy has been considered a viable therapy for patients with osteoporosis and explored, while no consensus on the clinically relevant oxidative stress‐related biomarkers for osteoporosis have been reached (Lee et al., 2021). A direct estimation of ROS levels is difficult due to their instability and short half‐life (Kim et al., 2015). Therefore, the assessment of oxidative stress is generally performed in an indirect way according to levels of intracellular ROS, mitochondrial ROS, and the activity of antioxidative enzymes as mitochondria serve as a major source of ROS (Wang et al., 2019). Chen et al. reported the significantly increased ROS both in cell and mitochondria after H_2_O_2_ treatment, accompanied by the markedly decreased indicators of osteogenesis, and the inhibited osteogenic differentiation and mineralization (Chen et al., 2021). In addition, Song et al. proposed a strong association of the decreased GPX and CAT activities with ROS (Zhu et al., 2017). Consistent with the above findings, Corynoline obviously alleviated H_2_O_2_‐induced oxidative stress and mitochondrial dysfunction in osteoblasts in the present study. Furthermore, Corynoline reversed the high apoptosis rate of H_2_O_2_‐treated cells. These outcomes indicated that Corynoline might exert protective effects on osteoblasts by attenuating oxidative stress levels. On the other hand, oxidative stress will inhibit the osteoblast differentiation that cause the accelerated bone loss. COL1A1, RUNX2, and BMP2 can be used as the markers of osteogenic differentiation, which were shown decreased with H_2_O_2_ treatment, with osteogenic phenotype loss of ALP and ARS. However, Corynoline could reversely upregulate their expression, restoring osteogenesis in vitro and effectively inhibiting bone microstructure damage caused by oxidative stress.

Nrf2 serves as an important transcription factor can enhance the expression of many antioxidative enzymes, including HO‐1(Do et al., 2021; Su et al., 2020), of which the signaling pathway plays an essential role in ROS scavenging and oxidative stress attenuation. Li et al. have reported the reversed osteoarthritis development by Corynoline via the Nrf2/NF‐kB pathway (Li et al., 2022). Liu et al. revealed that Corynoline attenuates LPS‐induced acute lung injury in mice by activating Nrf2, which was consistent with our findings that Corynoline supplementation induced the upregulated expression of the antioxidative protein HO‐1 and ROS removal by promoting the nuclear translocation of Nrf2(Li et al., 2022; Liu, Song, et al., 2017). Furthermore, we demonstrated the decreased Nrf2 expression by ML385, followed by the significantly weakened efficacy of Corynoline. Wu et al. proposed that Corynoline activated the Nrf2 pathway by promoting the phosphorylation of AKT and GSK3β (Wu et al., 2021). Liu et al. reported that Corynoline exerted its effects by manipulating the Nrf2 pathway and suppressing NF‐kB activation (Liu et al., 2018). Actually, the detailed mechanisms lying between Corynoline and Nrf2/HO‐1 requires the further investigation. Conclusively, the findings in the present study for the first time demonstrated that Corynoline could upregulate HO‐1 expression and Nrf2 activity to attenuate osteoporosis.

## 5 CONCLUSION

In summary, the present study revealed that Corynoline could upregulate the antioxidative enzyme expression in H2O2‐treated osteoblasts by activating the Nrf2 pathway, alleviating the negative effect of oxidative stress, and enhancing the function of osteoblasts. The in vitro experiment results showed that Corynoline delayed osteoporosis progression and restored the Nrf2 in situ expression. Overall, Corynoline could activate the Nrf2 pathway and mitigate or suppress the osteoporosis‐related bone loss through the Nrf2 pathway, which is expected to serve as a potential treatment for osteoporosis.

## AUTHOR CONTRIBUTIONS

All authors listed made substantial contributions to the study. Bing‐li Bai, Wen‐lai Fang; Tian‐Hao Xu, She‐ji Weng participated in the experimental design and contributed to reagents, materials, and analytical tools. Bing‐hao Lin, Jing‐yu Sun, and Kai Tan were also involved in the experiments. Bing‐li Bai, Tian‐hao Xu, and Wei‐kai Chen wrote the manuscript. Wen‐lai Fang, Cheng‐bin Huang, Run‐xun Ma, and Yi‐xun Huang performed data analysis. All authors read and approved the final version of the manuscript. All data shown in the figure are from the above authors' experiments.

## CONFLICT OF INTEREST

There were no financial or other conflicts of interest in designing, performing, or drafting this work.

## Data Availability

The data used to support the findings of this study are available from the corresponding author upon request.

## References

[fsn33239-bib-0001] Chen, L. , Hu, S. L. , Xie, J. , Yan, D. Y. , Weng, S. J. , Tang, J. H. , Wang, B. Z. , Xie, Z. J. , Wu, Z. Y. , & Yang, L. (2020). Proanthocyanidins‐mediated Nrf2 activation ameliorates glucocorticoid‐induced oxidative stress and mitochondrial dysfunction in osteoblasts. Oxidative Medicine and Cellular Longevity, 2020, 9102012. 33062149 10.1155/2020/9102012 PMC7533007

[fsn33239-bib-0002] Chen, L. , Shi, X. , Xie, J. , Weng, S. J. , Xie, Z. J. , Tang, J. H. , Yan, D. Y. , Wang, B. Z. , Fang, K. H. , Hong, C. X. , Wu, Z. Y. , & Yang, L. (2021). Apelin‐13 induces mitophagy in bone marrow mesenchymal stem cells to suppress intracellular oxidative stress and ameliorate osteoporosis by activation of AMPK signaling pathway. Free Radical Biology & Medicine, 163, 356–368. 33385540 10.1016/j.freeradbiomed.2020.12.235

[fsn33239-bib-0003] Do, N. Q. , Zheng, S. , Park, B. , QTN, N. , Choi, B. R. , Fang, M. , Kim, M. , Jeong, J. , Choi, J. , Yang, S. J. , & Yi, T. H. (2021). Camu‐Camu fruit extract inhibits oxidative stress and inflammatory responses by regulating NFAT and Nrf2 signaling pathways in high glucose‐induced human keratinocytes. Molecules, 26(11), 3174. 34073317 10.3390/molecules26113174 PMC8198278

[fsn33239-bib-0004] Khan, M. N. , & Khan, A. A. (2008). Cancer treatment‐related bone loss: a review and synthesis of the literature. Current Oncology (Toronto, Ont), 15(Suppl 1), S30–S40. 18231646 10.3747/co.2008.174 PMC2216420

[fsn33239-bib-0005] Kim, H. , Hong, M. K. , Choi, H. , Moon, H. S. , & Lee, H. J. (2015). Chemopreventive effects of korean red ginseng extract on rat hepatocarcinogenesis. Journal of Cancer, 6(1), 1–8. 25553083 10.7150/jca.10353 PMC4278909

[fsn33239-bib-0006] Kuek, V. , Yang, Z. , Chim, S. M. , Zhu, S. , Xu, H. , Chow, S. T. , et al. (2016). NPNT is expressed by osteoblasts and mediates angiogenesis via the activation of extracellular signal‐regulated kinase. Scientific Reports, 6, 36210. 27782206 10.1038/srep36210 PMC5080588

[fsn33239-bib-0007] Lee, C. W. , Lin, H. C. , Wang, B. Y. , Wang, A. Y. , Shin, R. L. , Cheung, S. Y. L. , & Lee, O. K. (2021). Ginkgolide B monotherapy reverses osteoporosis by regulating oxidative stress‐mediated bone homeostasis. Free Radical Biology Medicine, 168, 234–246. 33781894 10.1016/j.freeradbiomed.2021.03.008

[fsn33239-bib-0008] Li, S. , Shi, Y. , Zhang, S. , Li, H. , Ye, Z. , Kong, J. , Hong, W. , Tu, Y. , Ren, J. , Meftah, Z. , & Xie, C. (2022). Corynoline alleviates osteoarthritis development via the Nrf2/NF‐κB pathway. Oxidative Medicine and Cellular Longevity, 2022, 2188145. 35941903 10.1155/2022/2188145 PMC9356246

[fsn33239-bib-0009] Liu, B. , Su, K. , Wang, J. , Wang, J. , Xin, Z. , Li, F. , & Fu, Y. (2018). Corynoline exhibits anti‐inflammatory effects in lipopolysaccharide (LPS)‐stimulated human umbilical vein endothelial cells through activating Nrf2. Inflammation, 41(5), 1640–1647. 29748730 10.1007/s10753-018-0807-6

[fsn33239-bib-0010] Liu, W. , Mao, L. , Ji, F. , Chen, F. , Hao, Y. , & Liu, G. (2017). Targeted activation of AMPK by GSK621 ameliorates H2O2‐induced damages in osteoblasts. Oncotarget, 8(6), 10543–10552. 28060740 10.18632/oncotarget.14454 PMC5354679

[fsn33239-bib-0011] Liu, Y. , Song, M. , Zhu, G. , Xi, X. , Li, K. , Wu, C. , & Huang, L. (2017). Corynoline attenuates LPS‐induced acute lung injury in mice by activating Nrf2. International Immunopharmacology, 48, 96–101. 28486213 10.1016/j.intimp.2017.04.029

[fsn33239-bib-0012] National Research Council Committee for the Update of the Guide for the C, Use of Laboratory A . (2011). *The National Academies Collection: Reports funded by National Institutes of Health*. *Guide for the Care and Use of Laboratory Animals* . Washington (DC): National Academies Press (US) Copyright © 2011, National Academy of Sciences.

[fsn33239-bib-0013] Ni, L. , Lin, Z. , Hu, S. , Shi, Y. , Jiang, Z. , Zhao, J. , Zhou, Y. , Wu, Y. , Tian, N. , Sun, L. , & Wu, A. (2022). Itaconate attenuates osteoarthritis by inhibiting STING/NF‐κB axis in chondrocytes and promoting M2 polarization in macrophages. Biochemical Pharmacology, 198, 114935. 35104478 10.1016/j.bcp.2022.114935

[fsn33239-bib-0014] Park, P. S. U. , Mun, S. H. , Zeng, S. L. , Kim, H. , Bae, S. , & Park‐Min, K. H. (2020). Nrf2 is an upstream regulator of MYC‐mediated osteoclastogenesis and pathological bone erosion. Cells, 9(9), 2133. 32967239 10.3390/cells9092133 PMC7564846

[fsn33239-bib-0015] Sahni, S. , Hannan, M. T. , Blumberg, J. , Cupples, L. A. , Kiel, D. P. , & Tucker, K. L. (2009). Protective effect of total carotenoid and lycopene intake on the risk of hip fracture: a 17‐year follow‐up from the Framingham Osteoporosis Study. Journal of Bone and Mineral Research, 24(6), 1086–1094. 19138129 10.1359/JBMR.090102 PMC2683648

[fsn33239-bib-0016] Squillaro, T. , Alessio, N. , Capasso, S. , Di Bernardo, G. , Melone, M. A. B. , Peluso, G. , et al. (2019). Senescence phenomena and metabolic alteration in mesenchymal stromal cells from a mouse model of rett syndrome. International Journal of Molecular Science, 20(10), 2508. 10.3390/ijms20102508 PMC6567034 31117273

[fsn33239-bib-0017] Su, L. , Cao, P. , & Wang, H. (2020). Tetrandrine mediates renal function and redox homeostasis in a streptozotocin‐induced diabetic nephropathy rat model through Nrf2/HO‐1 reactivation. Annals of Translational Medicine, 8(16), 990. 32953790 10.21037/atm-20-5548 PMC7475465

[fsn33239-bib-0018] Sunthonkun, P. , Palajai, R. , Somboon, P. , Suan, C. L. , Ungsurangsri, M. , & Soontorngun, N. (2019). Life‐span extension by pigmented rice bran in the model yeast Saccharomyces cerevisiae. Scientific Reports, 9(1), 18061. 31792269 10.1038/s41598-019-54448-9 PMC6888876

[fsn33239-bib-0019] Wang, P. , Deng, J. , Dong, J. , Liu, J. , Bigio, E. H. , Mesulam, M. , et al. (2019). TDP‐43 induces mitochondrial damage and activates the mitochondrial unfolded protein response. PLoS Genetics, 15(5), e1007947. 31100073 10.1371/journal.pgen.1007947 PMC6524796

[fsn33239-bib-0020] Wei, L. , Ren, D. , Zhao, G. , & Zhao, L. (2021). Protective effect of corynoline in a murine allergic rhinitis model via inhibition of caspase‐1/NF‐κB. Archiv der Pharmazie, 354(2), e2000231. 33124097 10.1002/ardp.202000231

[fsn33239-bib-0021] Wu, Y. , He, T. , Fu, Y. , & Chen, J. (2021). Corynoline protects lipopolysaccharide‐induced mastitis through regulating AKT/GSK3β/Nrf2 signaling pathway. Environmental Toxicology, 36(12), 2493–2499. 34477289 10.1002/tox.23362

[fsn33239-bib-0022] Xiao, L. , Zhong, M. , Huang, Y. , Zhu, J. , Tang, W. , Li, D. , Shi, J. , Lu, A. , Yang, H. , Geng, D. , Li, H. , & Wang, Z. (2020). Puerarin alleviates osteoporosis in the ovariectomy‐induced mice by suppressing osteoclastogenesis via inhibition of TRAF6/ROS‐dependent MAPK/NF‐κB signaling pathways. Aging, 12(21), 21706–21729. 33176281 10.18632/aging.103976 PMC7695364

[fsn33239-bib-0023] Yeh, P. S. , Chen, J. T. , Cherng, Y. G. , Yang, S. T. , Tai, Y. T. , & Chen, R. M. (2020). Methylpiperidinopyrazole attenuates estrogen‐induced mitochondrial energy production and subsequent osteoblast maturation via an estrogen receptor alpha‐dependent mechanism. Molecules (Basel, Switzerland), 25(12), 2876. 32580515 10.3390/molecules25122876 PMC7356510

[fsn33239-bib-0024] Zhu, S. , Luo, F. , Zhu, B. , & Wang, G. X. (2017). Mitochondrial impairment and oxidative stress mediated apoptosis induced by α‐Fe(2)O(3) nanoparticles in Saccharomyces cerevisiae. Toxicology Research, 6(5), 719. 30090539 10.1039/c7tx00123a PMC6062213

